# FGF Signaling Regulates the Number of Posterior Taste Papillae by Controlling Progenitor Field Size

**DOI:** 10.1371/journal.pgen.1002098

**Published:** 2011-06-02

**Authors:** Camille I. Petersen, Andrew H. Jheon, Pasha Mostowfi, Cyril Charles, Saunders Ching, Shoba Thirumangalathu, Linda A. Barlow, Ophir D. Klein

**Affiliations:** 1 Department of Orofacial Sciences and Program in Craniofacial and Mesenchymal Biology, University of California San Francisco, San Francisco, California, United States of America; 2 Team “Evo-Devo of Vertebrate Dentition,” Institut de Génomique Fonctionnelle de Lyon, Université de Lyon, CNRS, Ecole Normale Supérieure de Lyon, Lyon, France; 3 Department of Cell and Developmental Biology and the Rocky Mountain Taste and Smell Center, University of Colorado School of Medicine, Denver, Colorado, United States of America; 4 Department of Pediatrics and Institute for Human Genetics, University of California San Francisco, San Francisco, California, United States of America; University of Helsinki, Finland

## Abstract

The sense of taste is fundamental to our ability to ingest nutritious substances and to detect and avoid potentially toxic ones. Sensory taste buds are housed in papillae that develop from epithelial placodes. Three distinct types of gustatory papillae reside on the rodent tongue: small fungiform papillae are found in the anterior tongue, whereas the posterior tongue contains the larger foliate papillae and a single midline circumvallate papilla (CVP). Despite the great variation in the number of CVPs in mammals, its importance in taste function, and its status as the largest of the taste papillae, very little is known about the development of this structure. Here, we report that a balance between Sprouty (Spry) genes and *Fgf10*, which respectively antagonize and activate receptor tyrosine kinase (RTK) signaling, regulates the number of CVPs. Deletion of *Spry2* alone resulted in duplication of the CVP as a result of an increase in the size of the placode progenitor field, and *Spry1^−/−^*;*Spry2^−/−^* embryos had multiple CVPs, demonstrating the redundancy of Sprouty genes in regulating the progenitor field size. By contrast, deletion of *Fgf10* led to absence of the CVP, identifying FGF10 as the first inductive, mesenchyme-derived factor for taste papillae. Our results provide the first demonstration of the role of epithelial-mesenchymal FGF signaling in taste papilla development, indicate that regulation of the progenitor field size by FGF signaling is a critical determinant of papilla number, and suggest that the great variation in CVP number among mammalian species may be linked to levels of signaling by the FGF pathway.

## Author Summary

The sense of taste is important for an animal's ability to survive and thrive, because it enables discrimination between nutritious substances and toxins. Taste buds are housed largely on the tongue in structures called papillae; of the three types of gustatory papillae, the circumvallate papilla (CVP) is the largest. In rodents, a single CVP is located in the posterior midline of the tongue housing hundreds of taste buds, whereas in other mammals up to dozens of CVPs can be found. However, despite the great variation in the number of CVPs in mammals, its status as the largest of the taste papillae, and its importance in taste function, very little is known about its development. We identified members of the FGF signaling pathway as determinants of CVP number. We propose that perturbations to the FGF signaling pathway may have been involved in the dramatic differences in CVP number that arose during mammalian evolution.

## Introduction

**Figure 1:**
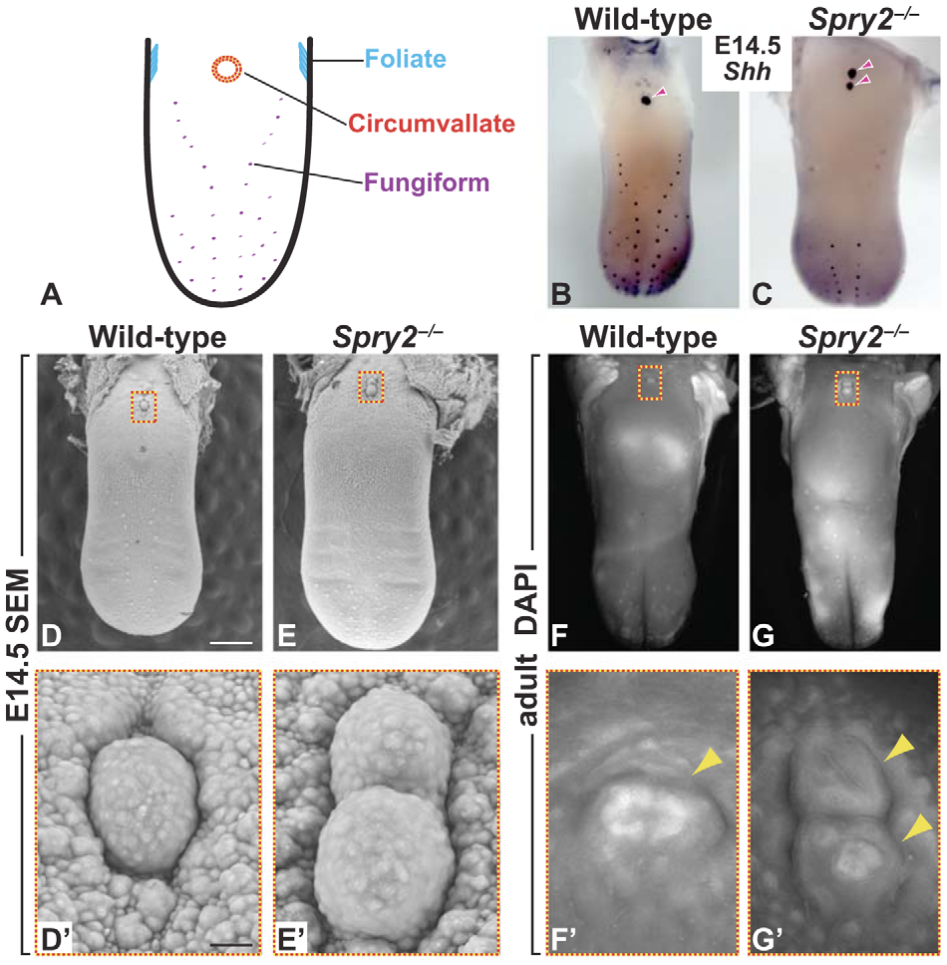
Deletion of *Spry2* leads to a duplication of the CVP in the posterior tongue. (A) Cartoon showing location of gustatory papillae in the rodent tongue. (B,C) *In situ* hybridization staining of *Shh.* Wild-type mice possess a single CVP in the posterior tongue, whereas the CVP is duplicated in *Spry2^−/−^* mice (*arrowheads*). (D,E) SEM images of the tongue at E14.5. Scale bar, 500 µm. (F,G) DAPI fluorescence staining shows that the two CVPs (*arrowheads*) persist into adulthood in *Spry2^−/−^* mice. (D'-G') Higher magnification images of boxed areas. (D',E') Scale bar, 25 µm.

Taste sensory capability is mediated by aggregates of receptor cells, called taste buds, which reside within the oral and pharyngeal cavities. The majority of taste buds in mammals reside on the tongue in epithelial-mesenchymal specializations termed gustatory papillae. In the rodent tongue, the smaller fungiform papillae, each of which possesses a single taste bud, are found in a distributed array on the anterior tongue. By contrast, the larger bilateral foliate papillae and a single midline circumvallate papilla (CVP) each house hundreds of buds and reside on the posterior tongue (Figure 1A). Recently, there has been increasing recognition that anterior fungiform taste buds differ from those of the posterior CVP in terms of both gene expression and taste function [1]–[4].

In recent years, significant progress has been made in defining the molecular regulation of fungiform development. Fungiform papillae initially form as placodes that subsequently undergo epithelial morphogenesis and acquire a mesenchymal core [5] in a process that is similar to morphogenesis of other vertebrate epithelial specializations, such as hair, teeth, and mammary glands [6], [7]. The development of these other organs requires signaling between epithelium and mesenchyme, suggesting that such epithelial-mesenchymal interactions are also involved in patterning and morphogenesis of taste placodes. However, expression of all of the key signaling factors implicated in taste placode development – including Sonic Hedgehog (SHH), Bone Morphogenetic Proteins (BMPs), Epidermal Growth Factor (EGF), and WNTs – is restricted to the epithelium [8]–[15]. Thus, to date, no inductive, mesenchyme-derived factor involved in taste development has been identified.

Despite its importance in taste function and its status as the largest of the taste papillae, very little is known about the genes involved in the development of the CVP. Like fungiform placodes, the CVP forms as an initial epithelial thickening that undergoes complex morphogenesis to form a large papilla. However, it appears that genes known to regulate fungiform development do not function similarly in development of the CVP. For example, inhibition of SHH results in more and larger fungiform placodes, but has no effect on the CVP [11]. In addition, BMP7 and its antagonist follistatin have significant functions in fungiform development, but the CVP appears unaffected by inactivation of either gene [16]. These differences may be ascribed to the distinct embryonic origins of the anterior tongue, which is thought to be derived from ectoderm, whereas the posterior tongue likely has endodermal origins [4].

Expression of several Fibroblast Growth Factors (FGFs) and their receptors has previously been detected in the developing tongue [17]. Therefore, we hypothesized that Sprouty (Spry) genes, which antagonize several receptor-tyrosine kinase (RTK) signaling pathways including those triggered by FGFs [18]–[20], may play a role in the development of taste papillae. Originally, *spry* was identified as a regulator of tracheal branching in *Drosophila* [21], and it was later found that three of the four mouse Sprouty genes (*Spry1*, *Spry2*, and *Spry4*) are expressed during embryonic development [22], [23].

Here, we used mouse genetic models to show that FGF signaling is required for CVP formation. We found that *Spry1* and *Spry2* antagonize signaling by FGF10 to restrict the size of the progenitor field of the circumvallate (CV) placode, such that loss of Sprouty function results in a dramatic expansion of the CV placode as it first forms. In adult *Spry2* mutants, a striking and complete duplication of the CVP emerges, whereas embryos lacking both *Spry1* and *Spry2* have multiple CVPs. Our findings thus represent the first example of molecular genetic regulation of taste organs in the posterior tongue. We found that *Fgf10*, which is expressed exclusively in the mesenchyme underlying the CV placode, is required for formation of the CVP, and thus we provide the first example of an inductive, mesenchymal signal involved in specifying the epithelial domain of a developing taste papilla. Further, while FGF10 signaling drives CVP development and *Spry1* and *Spry2* repress this process, fungiform taste papillae are oppositely affected by the loss of these genes: *Fgf10* ^−/−^ tongues appear to possess more and larger fungiform papillae, and the loss of Sprouty genes results in fewer fungiform papillae. Thus, these results demonstrate that molecular mechanisms regulating development of anterior and posterior taste organs differ considerably. Finally, we postulate that the role of FGF signaling in defining the size of the CV progenitor field in mice may underlie the large variation in CVP number across mammalian taxa, and that changes in FGF signaling during evolution may have caused expansion of the initial progenitor field to allow formation of multiple CVPs in some species.

## Results

### Deletion of *Spry2* leads to a duplication of the CVP

Wild-type mice possess a single CVP in the midline of the posterior tongue; foliate papillae reside on the lateral tongue and multiple fungiform papillae populate the anterior tongue (Figure 1A, 1B). In *Spry2* ^−/−^ mice, we observed a duplication of the CVP in the anterior-posterior orientation at embryonic day (E) 14.5 using *Shh* expression as a marker (Figure 1C). By contrast, fungiform placodes in the anterior tongue, which also express *Shh*, were significantly reduced in number (61±4.37 in wild-type versus 33±2.02 in *Spry2* ^−/−^ embryos; p = 0.0002; n = 6 embryos per genotype). Because alterations in fungiform papilla development had been reported in other mutants [9]–[15], we chose to pursue the novel CVP duplication phenotype. The presence of two CVPs in the embryonic tongue was confirmed using scanning electron microscopy (SEM; Figure 1D-1E'). To test whether the duplicated CVP persisted into adulthood, we used DAPI staining in adult tongues and again identified two discrete papillae (Figure 1F-1G'). Notably, both CVPs in *Spry2* ^−/−^ mice house fully differentiated taste buds containing the three types of taste receptor cells, and both showed positive staining for b3-tubulin, indicating that taste buds and the papillae are innervated (Figure S1). Taken together, these data indicate that the duplication in *Spry2* ^−/−^ embryos leads to two functional adult CVPs.

**Figure 2:**
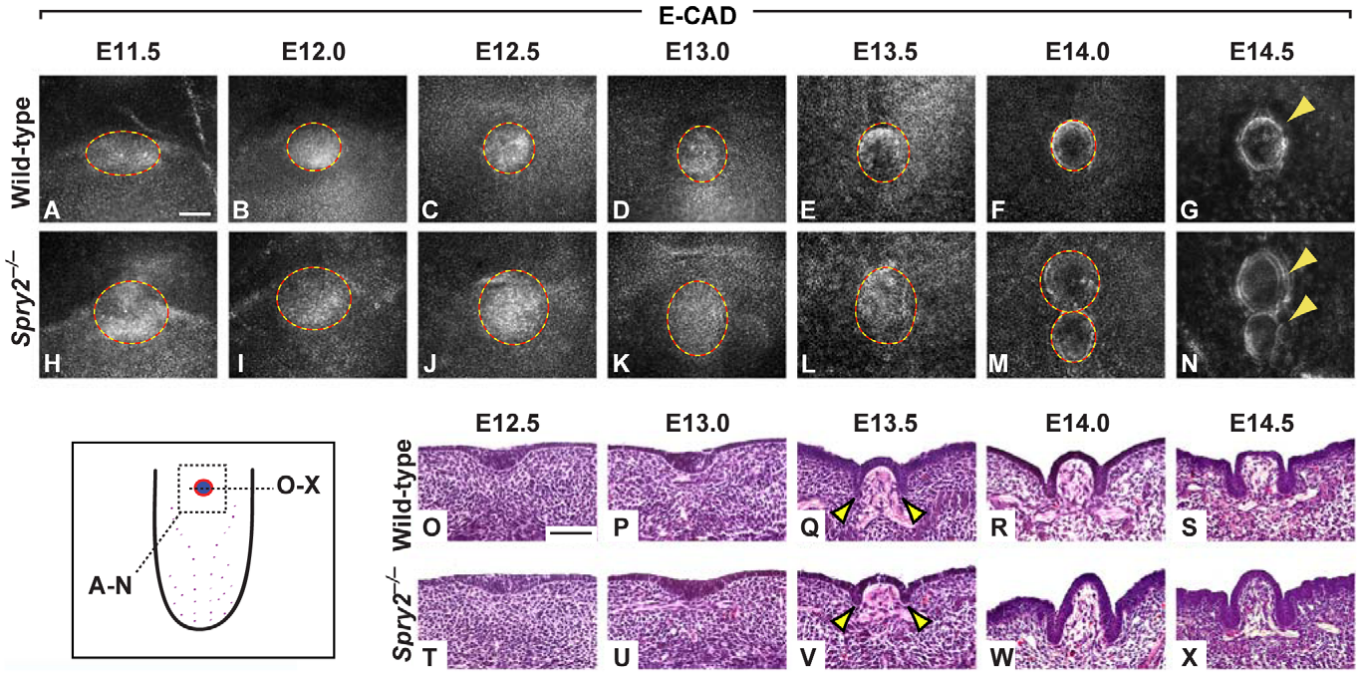
Development of the CVP in wild-type and *Spry2^−/−^* mice. (Inset) Cartoon depicting the planes of observation. (A–N) E-cadherin (E-CAD) immunofluorescence staining of CV placodes and papillae in wild-type (A–G) and *Spry2^−/−^* (H-N) mice. (O–X) H&E stains of coronal sections of the single CV placode and papilla in wild-type and the proximal CV placode and papilla in *Spry2^−/−^* mice. The size of the papilla was quantified between E13 to E14 (D–F, K–M; ×10^−3^ um). *Arrowheads* indicate developing trenches. Scale bars, 50 µm.

To reconstruct the development of the CVP, closely staged specimens between E11.5 to E14.5 were stained with E-cadherin and imaged in whole-mount (Figure 2A-2N), as well as analyzed by H&E staining of sections (Figure 2O-2X). In wild-type embryos, CVP development was initiated shortly after the tongue rudiment formed by fusion of the lateral lingual swellings, and it was detected as a more compact collection of epithelial cells at E11.5 (Figure 2A) [24]. At E12.5, a placodal condensation was readily observable on coronal sections (Figure 2O). Between E13 and E13.5, the wild-type placode underwent major morphological changes as the epithelial trenches characteristic of the CVP began to form (Figure 2P, 2Q). The growth of the trenches was coincident with the initiation of innervation at approximately E13 (Figure S2), and the trenches continued to grow into the mesenchyme at E14.5 (Figure 2G, 2S) [24], [25].

In contrast to the wild-type, the CV placode in *Spry2^−/−^* embryos was larger at E11.5 (compare Figure 2A, 2H). Relative to the wild-type, the mutant placode continued to grow larger both laterally and along the anterior-posterior axis between E11.5 and E13.5 (Figure 2B-2E, 2I-2L). By E14, two distinct structures were observed in *Spry2* ^−/−^ embryos, with the anterior of the two papillae usually appearing smaller than the posterior at E14 and E14.5 (Figure 2M, 2N). In addition, CVPs in *Spry2* ^−/−^ embryos showed a raised dome shape compared to the flatter shape of the wild-type CVP (Figure 2R, 2S, 2W, 2X).

Several possible mechanisms could account for the duplication of the CVP at ∼E14, including a timer that causes the organ to split at a certain point after its development starts or a threshold that causes splitting after a critical size limit is achieved. To address this issue, we quantified the size of the CV placode and CVP in wild-type and *Spry2^−/−^* tongues between E13 to E14, corresponding to the time points before, during and after CVP duplication (Figure S2J). Relative to wild-type mice, the mutant CV placode was already significantly larger at E13 and E13.5, but when CVP duplication was observed at E14, there was a further and dramatic increase in the total area of the papillae. Thus, our data suggest that there is a critical threshold size for the CV placode, beyond which the placode destabilizes, leading to CVP duplication.

### The CV progenitor field is enlarged in *Spry2^−/−^* mice

**Figure 3:**
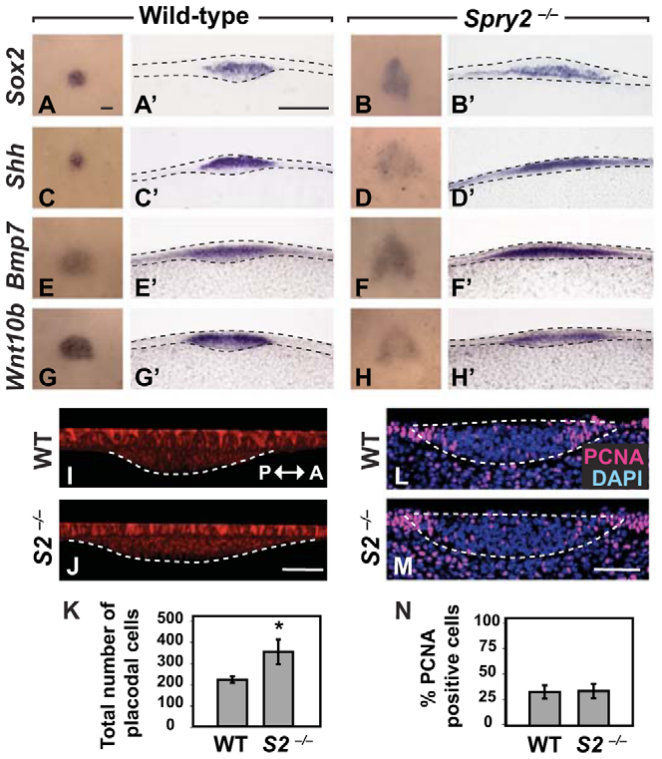
The CV progenitor field at E12.5 is expanded in *Spry2* ^−/−^ mice. (A–H) Dorsal view of the expression of *Sox2*, *Shh*, *Bmp7*, and *Wnt10b* in wild-type and *Spry2* ^−/−^ littermates. Scale bar, 50 µm. (A'–H') Coronal sections of A–H. Scale bar, 50 µm. (I,J) Sagittal views of the CV placodes stained with E-cadherin show an expansion of the placode in the anterior(A)-posterior(P) orientation in *Spry2* ^−/−^ mice. Scale bar, 20 µm. (K) Quantification of the total number of cells in the CV placode. (L,M) Proliferating cells were detected by PCNA staining in coronal view. Scale bar, 20 µm. (N) The percentage of PCNA-positive cells in the CV placode at E12.5. * p<0.01. WT, wild-type; *S2* ^−/−^, *Spry2* ^−/−^.

*Sox2* is one of the earliest markers of taste placodes and is required for taste bud development [26]. *Sox2* expression was expanded in the placode domain in both anterior-posterior and lateral directions in *Spry2* ^−/−^ embryos compared to wild-type littermates at both E11.5 (data not shown) and E12.5 (Figure 3A-3B'). Next, we analyzed the expression of three additional factors that mark the early placode: *Shh, Bmp7,* and *Wnt10b* (Figure 3C-3H'). *Shh* has previously been shown to be expressed in the developing CVP [9], [27], and in fungiform placodes, *Shh* is expressed specifically by taste bud progenitors [28]. *Bmp7* and *Wnt10b* have also been implicated in development of fungiform papillae [15], [16]. The expression domains of all these markers were expanded in the CV placode of *Spry2* ^−/−^ embryos relative to wild-type (Figure 3C-3H'). We quantified the differences in expression levels by qPCR, and interestingly, whereas *Sox2* and *Bmp7* levels were strongly upregulated in the mutants, consistent with the expanded expression domains seen in whole mount, *Shh* and *Wnt10b* expression levels were not dramatically different in the mutants (Figure S3A). This is consistent with the decreased intensity of *Shh* and *Wnt10b* staining despite an increased CV placodal domain size (Figure 3D, 3D', 3H, 3H').

We next asked whether there was a difference in the number of cells in the placode between wild-type and mutant. We quantified the number of cells in the CV placode of *Spry2* ^−/−^ embryos relative to wild-type littermates at E12.5 from three-dimensional confocal images of E-cadherin-positive stained cells (Figure 3I-3K). We counted cells in 10 mm intervals beginning at the deepest tip of invaginated cells using ventral views; the borders of the placode were defined by the invagination. This analysis showed that already by the mid-placode stage, the mutant placode had almost twice as many cells as the wild-type. Because differences in proliferation or cell death could account for the larger placode and the morphogenetic abnormalities in the CVPs of *Spry2* mutants, we assayed proliferation by PCNA staining (Figure 3L-3N), and cell death by TUNEL staining (Figure S3B-S3D). We detected no differences between wild-type and mutant CV placode at E12.5 for either assay, suggesting that a larger number of cells was recruited into the placode at the earliest stages of placodogenesis. Thus, our data indicate that inactivation of *Spry2* leads to a larger progenitor field, as detected by multiple molecular markers, such that the duplication in the *Spry2* mutant results from recruitment of more cells into the CV placode at the earliest stages of its development.

### *Spry1* and *Spry2* are co-expressed in the CV placode, and hypersensitivity to FGF signaling is detected in *Spry2* mutants

**Figure 4:**
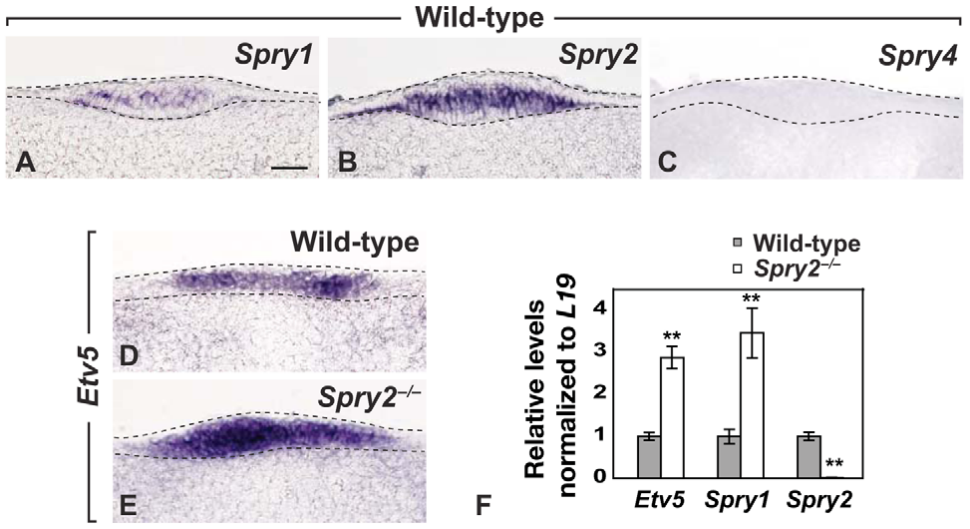
*Spry1* and *Spry2* are co-expressed in the CV placode and increased FGF signaling is detected in *Spry2* mutants. (A–C) Expression of *Spry1*, *Spry2*, and *Spry4* in the CV placode of wild-type mice. (D,E) Expression of *Etv5* in the CV placode of wild-type and *Spry2* ^−/−^ mice. (F) qPCR analysis of the expression profiles of *Etv5* and Sprouty genes in the posterior tongue of wild-type and *Spry2* ^−/−^ mice at E12.5. Scale bar, 20 µm. **, p<0.001.

Because Sprouty genes are often co-expressed during development [23], [29], we assayed for expression of *Spry1*, *Spry2*, and *Spry4* in CV placodes at E12.5 (Figure 4A-4C); *Spry3* was not analyzed due to its lack of expression in embryonic craniofacial tissues [23]. We found that *Spry1* and *Spry2* were both expressed in the epithelium of the CV placode at E12.5, whereas no expression was observed for *Spry4* (Figure 4A-4C).

The expression of *Etv5* (*Erm*), which is a target of FGF signaling [30], was upregulated in *Spry2* ^−/−^ embryos (compare Figure 4D, 4E), consistent with increased FGF signaling observed with Sprouty loss-of-function in other developmental contexts [31]–[34]. To quantify expression levels, RNA was extracted from the area containing the CV placode at E12.5 and analyzed by qPCR, and a three-fold increase in *Etv5* was observed (Figure 4F). As expected, essentially no *Spry2* expression was detected in the *Spry2* nulls, but interestingly, *Spry1* expression was dramatically increased in *Spry2* ^−/−^ mice (Figure 4F). This upregulation is consistent with the known role of Sprouty genes as FGF targets [21], [23], and it suggested that increased expression of *Spry1* may serve a compensatory role in *Spry2* ^−/−^ mice. qPCR showed that *Spry4* was expressed at very low levels, with no detectable difference between wild-type and *Spry2* ^−/−^ littermates (data not shown).

### *Fgf10* is required for CVP development and is antagonized by *Spry2*

**Figure 5:**
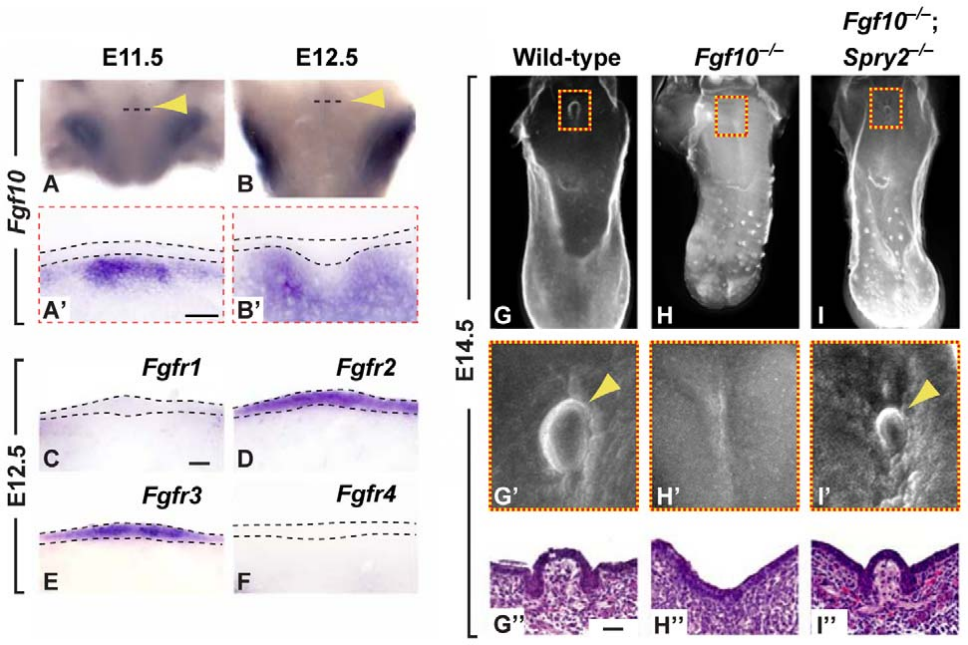
*Fgf10* is required for CVP development and is antagonized by *Spry2*. (A–B') Expression of *Fgf10* in the CV placode of wild-type mice (arrowhead). Dashed line depicts the coronal plane of section for A' and B'. (C–F) Expression of FGF receptors (*Fgfr1-4*) in coronal sections. (G–I”) Genetic rescue of the loss of CVP phenotype in *Fgf10^−/−^* mice (H) by deletion of *Spry2* (I) as visualized by E-cadherin immunofluorescence staining. (G'–I') Higher magnification views of boxed areas. (G”–I”) H&E staining of coronal sections through the CVP. Scale bars, 25 µm.

The epithelial expression of *Spry1*, *Spry2*, and *Etv5*, all of which are expressed in response to RTK activity [21], [23], [30], strongly suggested the presence of a mesenchymal RTK ligand that signals to the epithelium. To identify such a ligand, we surveyed the expression of a number of candidate RTKs and their cognate ligands by ISH, including *Fgfr1-4, Egfr*, *Fgf7*, *Fgf8*, *Fgf9*, and *Fgf10* (data not shown). Of the ligands we examined, only *Fgf10* was expressed in the mesenchyme subjacent to the epithelium along the midline of the posterior tongue at E11.5 and E12.5 (Figure 5A-5B'). Two FGF receptors, *Fgfr2* and *Fgfr3,* were also expressed in the CV placode, whereas no expression of *Fgfr1* or *Fgfr4* was detected (Figure 5C-5F).

To further test the role of FGF signaling in CVP development, we isolated tongues from wild-type embryos at E11.5 and E12.5 and grew them *in vitro* either in the absence or presence of SU5402 [35], a potent FGF receptor inhibitor (Figure S4). In the absence of SU5402, we found the formation of a single CVP. The inhibition of FGF signaling by SU5402 led to the absence of the CVP in tongues cultured at E11.5 and to either an absent or a malformed CVP in tongues cultured at E12.5. These observations demonstrate the requirement of FGF signaling during CVP formation at E11.5 and to a lesser extent at E12.5.

Because *Fgf10* was expressed directly underneath the developing CV placode, we hypothesized that the ligand encoded by this gene may play a role in CV development, and therefore we examined the CVP in *Fgf10* ^−/−^ embryos. Strikingly, the deletion of *Fgf10* led to the complete absence of the CVP (Figure 5H-5H”), indicating that this gene plays a critical inductive role in development of this structure. Next, we reasoned that the combined deletion of *Fgf10* and *Spry2* may balance each other and rescue CVP development, and indeed, we found a single CVP in *Fgf10^−/−^*;*Spry2^−/−^* mice (Figure 5I-5I”). This result also implies the presence of a yet-unknown secondary ligand that can fulfill the function of FGF10 if the epithelium is rendered hypersensitive to RTK signaling by inactivation of *Spry2.* Taken together, our data indicate that the FGF signaling pathway is critical for proper CVP development and that it determines the final number of CVPs.

### Combined deletion of *Spry1* and *Spry2* leads to multiple CVPs

**Figure 6:**
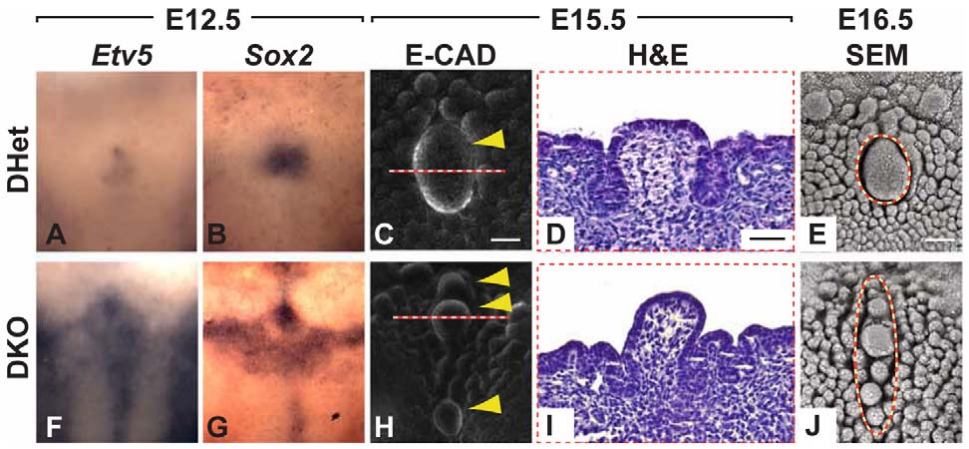
Formation of multiple CVPs in *Spry1^−/−^*;*Spry2^−/−^* (DKO) mice. (A,B,F,G) Dorsal views of the expression patterns of *Etv5* and *Sox2* in *Spry1^+/−^*;*Spry2^+/−^* (DHet) and DKO mice. (C,H) Multiple CVPs in the anterio-posterior direction were observed in DKO mice visualized by E-cadherin immunofluorescence staining (*arrowheads*). Scale bar, 50 µm. (D,I) H&E staining of coronal sections through the CVP as indicated by dashed lines in C and H. Scale bar, 25 µm. (E,J) Multiple, presumptive CVPs visualized by SEM are indicated by dashed circles. Scale bar, 50 µm.

Based on the overlapping expression of *Spry1* and *Spry2* in the CV placode and the upregulation of *Spry1* in *Spry2^−/−^* embryos (Figure 4F), we hypothesized that these two family members may be partially redundant. We therefore generated and analyzed *Spry1^−/−^* as well as *Spry1^−/−^*;*Spry2^−/−^* (DKO) embryos. No difference in the number or morphology of the CVP was detected in *Spry1^−/−^* embryos (data not shown), indicating that *Spry1* is not required for CVP development if *Spry2* is present. In contrast, CVPs from DKO embryos were dramatically abnormal relative to control *Spry1^+/−^*;*Spry2^+/−^* (DHet) littermates, whose tongues were indistinguishable from those of wild-types both as embryos and as adults (data not shown). At E12.5, the expression domains of *Etv5* and *Sox2* were expanded in the CV placodes of DKO mice relative to DHet littermates (Figure 6A, 6B, 6F, 6G). The expanded expression domains in the DKOs were greater than those seen in *Spry2^−/−^* mice (compare to Figure 4D, 4E and Figure 3A-3B'). At later stages, in contrast to the single duplication seen in *Spry2^−/−^* mice, the DKO embryos had multiple CVPs (Figure 6C, 6E, 6H, 6J). The multiple CVPs in DKO embryos were smaller than the single CVP in controls and showed pronounced raised domes (Figure 6D, 6I) similar to *Spry2^−/−^* embryos (Figure 2S, 2X). Immunofluorescence staining of β3-tubulin revealed innervation of the multiple CVPs (Figure S5).

## Discussion

Of the numerous gustatory papillae, very little is known about the development of the CVP, despite the great variation in CVP number in mammals, its importance in taste function, and its status as the largest of the taste papillae. Most of the studies on gustatory organ and taste development have focused on fungiform papillae, and it is probably for this reason that significant genetic abnormalities of the CVP have not yet been reported in mice. Here, we report several findings: first, FGF signaling plays a key role in the regulation of taste papilla development; second, FGF10 is an inductive, mesenchyme-derived factor required for CVP development; third, the regulation of the progenitor field size by Sprouty genes is a critical determinant of CVP number. Finally, we postulate that the great variation in CVP number among mammalian species may be linked to alterations in the genetic dosage of agonists and antagonists in the FGF signaling pathway.

### Antagonism between mesenchymal FGF10 and epithelial Sprouty genes controls CVP number

Antagonism of FGF signaling by Sprouty genes occurs during development of a number of organs [31]–[34]. Because FGFs and their receptors had previously been detected in the developing tongue [17], we hypothesized that Sprouty genes, which modulate several RTK signaling pathways including those triggered by FGFs [18]–[20], may play a role in taste papillae development. Whereas deletion of *Spry2* led to a duplication of the CVP, *Fgf10^−/−^* mice showed a complete loss of the CVP, and importantly, CVP development was rescued in compound *Spry2^−/−^*; *Fgf10^−/−^* mutants. These results confirm the specific antagonism of FGF10 signaling by SPRY2 and establish the importance of the FGF signaling pathway in determining CVP number. Additionally, the results with the *Spry2^−/−^*;*Fgf10^−/−^* mutants indicate the presence of an unknown, secondary factor; such a factor could induce CVP formation in lieu of FGF10 when the epithelium is hypersensitive to RTK signaling due to the absence of *Spry2*.

Because of the overlapping expression profiles of *Spry1* and *Spry2*, and the upregulation of *Spry1* in the *Spry2^−/−^* CV placode, we generated *Spry1^−/−^*; *Spry2^−/−^* embryos. We observed multiple CVPs along the A-P axis in the compound mutant embryos, demonstrating the redundancy of Sprouty genes, for which there is precedent in other tissues [32], in regulating CVP development. Although a slightly dysplastic CVP was recently reported in ectodysplasin mutant mice [36], the phenotypes in *Fgf10^−/−^*, *Spry2^−/−^, and Spry1^−/−^*;*Spry2^−/−^* mice are the first genetic abnormalities reported involving the regulation of CVP number.

Despite indications that mesenchyme-derived factors are involved in lingual papillae development [5], [37], the only mesenchymal factor implicated in fungiform papillae formation to date is follistatin, an inhibitor of BMP signaling [16]. Therefore, FGF10 is the first inductive, mesenchyme-derived factor to be identified in taste papilla development.

### Expansion of the CV progenitor field

The duplication of the CVP at E14 in *Spry2^−/−^* embryos was preceded by an increase in placode size. The CV placode in mutant mice was significantly larger at E13 and E13.5 relative to wild-type, and at E14 there was a dramatic increase in total CVP size as duplication occurred (Figure S2J). Thus, there appears to be a critical size threshold for the CV placode, beyond which the placode destabilizes, resulting in splitting and duplication. Recently, Munne et al. [38] reported that, during incisor tooth development, the impairment of BMP4 signaling or an increase in Activin concentration led to the destabilization of the large, single placode and the formation of multiple incisors. Future experiments will enable comparison of the relative roles of different signaling pathways in maintenance of epithelial placode integrity.

The increase in placode size at E13 and E13.5 was preceded at E11.5 and E12.5 by the expansion of the expression domains for various genes, such as *Etv5*, *Sox2*, *Shh*, *Bmp7*, and *Wnt10b*. By E12.5, the mutant CV placode already had more cells than the control, and we did not detect any apoptosis in the CV placode or any differences in the percentage of proliferating cells. Thus, the larger CV placode in the *Spry2* mutant appears to result at least in part from an increase in the recruitment of CV placodal cells. At the earliest stages of placode formation, this could potentially occur either through specification of more placodal precursors or through migration of more cells into the placodal domain. Future studies will be needed to distinguish between these possibilities, but interestingly, several reports have pointed to a role for Sprouty genes in cell migration [39]–[41].

In the CV placode of *Spry1^−/−^*;*Spry2^−/−^* tongues, there were further increases in the expression domains of *Etv5* and *Sox2* beyond what was seen in the *Spry2^−/−^* alone, demonstrating the redundancy of *Spry1* and *Spry2* in this organ (Figure 6A, 6B, 6F, 6G). The compound deletion of *Spry1* and *Spry2* led to an even larger placode (Figure 6A, 6B, 6F, 6G), which resulted in more than two CVPs.

### Comparision of fungiform and CV papillae

Whereas the deletion of *Spry2* led to CVP duplication, it also resulted in a marked decrease in the number of fungiform papillae (Figure 1B, 1C). In contrast, in *Fgf10^−/−^*mice, there was an absence of the CVP, whereas the fungiform papillae appeared to be larger and more closely spaced (Figure 4G, 4H). Thus, *Fgf10* and Sprouty genes differentially affect the anterior versus posterior taste fields. Together with the previous studies showing effects of SHH and BMP7 on anterior but not posterior taste fields, these results provide further evidence for important developmental differences between these fields [9], [10], [12], [16]. This observation is consistent with the notion that fungiform papillae in the anterior tongue are derived from ectoderm, whereas the CVP is likely derived from endoderm [4]. The diverse responses of fungiform versus CV papillae to developmental factors have only recently been appreciated and will require further efforts to tease apart.

### Evolution of CVP number in mammals

Although most rodents, including the mouse, possess a single midline CVP in the posterior tongue, there is great variation in mammalian CVP number (Figure S6B, Figure S7). For example, humans possess anywhere from 3 to 14 CVPs in an inverted V or Y orientation [42]. Other mammals such as the hyrax [43], [44] and hippopotamus [45] possess no CVP, whereas siamang, chimpanzee, gorilla [46] and lemur [47] possess at least two CVPs along the midline, in addition to varying numbers of lateral CVPs (Figure S6A). We have shown that modifications in FGF signaling can lead to increased or decreased numbers of CVPs. Thus, we speculate that changes in levels of signaling through this pathway provide an attractive candidate for producing variation in CVP number, and in particular, for generating CVPs that are multiplied along the midline or anterior-posterior axis. It is interesting that both *Fgf10* and *Etv5*, an indicator of FGF signaling, were expressed along the midline, which correlates with anterior-posterior CVP multiplication in *Spry2^−/−^* and *Spry1^−/−^*;*Spry2^−/−^* mice.

The effect of CVP number on taste is currently unclear. Correlation between taste sensitivity and the number of taste buds within the CVP has been previously reported [48]. However, because there is variation in the number of taste buds per CVP, as well as in the number of taste cells within each taste bud [49], there is no clear indication of what the number of CVPs reveals about taste preferences. Whether the variation in mammalian CVP number provides evolutionary advantages in terms of identification of nutritious substances and detection and avoidance of potentially toxic ones remains to be elucidated.

In 1950, Spuhler [42] postulated that the variation in CVP number observed in humans was likely due to genetic factors involving at least 5 multiple alleles. Our studies indicate, for the first time, that the perturbation of a single gene such as *Fgf10* or *Spry2* may be sufficient to confer the vast genetic variation in mammalian CVP number.

## Materials and Methods

### Ethics statement

This study was carried out in strict accordance with the recommendations in the Guide for the Care and Use of Laboratory Animals of the National Institutes of Health. The protocol was approved by the UCSF Institutional Animal Care and Use Committee (Protocol Number: AN084146). All efforts were made to minimize animal suffering.

### Animals

Mouse lines carrying null or floxed alleles of *Spry1* [31], *Spry2* [34], and *Fgf10* [50] or β-actin Cre transgenes [51] were maintained and genotyped as described. *Spry1^−/−^*;*Spry2^−/−^* mutant embryos were generated by crossing β-actin Cre;*Spry1^+/−^*;*Spry2^+/−^* males with *Spry1^flox/flox^*;*Spry2^flox/flox^* females (double mutants produced at an expected Mendelian frequency of 1∶4). Tongues and taste papillae from double heterozygous embryos (*Spry1^+/−^*;*Spry2^+/−^*) were indistinguishable from wild-type CD-1 embryos and served as controls. Mice were mated overnight, and the presence of a vaginal plug indicated embryonic day (E) 0.5.

### Histology

Embryonic and adult tongues were fixed overnight in 4% paraformaldehyde at 4°C. For sections, tongues were dehydrated, embedded in paraffin wax, and serially sectioned at 7 µm. Histological sections were stained with haematoxylin and eosin (H&E).

### In situ hybridization (ISH)

Whole-mount ISH using digoxigenin-labeled RNA probes was performed according to standard protocols. RNA probes were generated from plasmids containing fragments of *Shh*, *Spry1*, *Spry2*, *Spry4*, *Fgf10*, and *Sox2* or from PCR-amplified fragments of *Wnt10b* and *Bmp7*. A 10 minute 2% H_2_O_2_ treatment was performed on tissues E14.5 and older. Stained specimens were incubated overnight in 30% sucrose, embedded in tissue freezing media (Triangle Biomedical Sciences, Durham, N.C.), and cryosectioned using a Leica CM1900 at 18 µm intervals.

### Immunohistochemistry (IHC)

Whole-mount IHC was performed according to a published protocol [52]. Rat anti-E-cadherin (1∶1000; Invitrogen Cat. 1300) or mouse anti-β3 tubulin (1∶250; R&D Systems) were applied followed by incubation in goat anti-rat AlexaFluor 555 secondary antibody (1∶250, Invitrogen). For β3-tubulin staining, the Mouse on Mouse kit (Vectastain) was used. Specimens were counterstained with DAPI. For IHC on sectioned embryonic specimens the same procedure was used after a rehydration step. For IHC on adult tongues, tissue was processed according to a published protocol [28] for markers of three types of taste receptor cells: type 1, rabbit anti-NTPdase2 (Nucleoside triphosphate diphosphohydrolase-2, 1∶1000) is the ecto-ATPase of type I cells in taste buds [53]; type II, monoclonal anti-IP3R3 (Receptor for inositol 1,4,5-trisphosphate, 1∶1000; BD Transduction) is a second messenger that mediates the release of intracellular calcium [54]; and type III, rabbit anti-NCAM (Neural cell adhesion molecule, 1∶1000) [55]. Secondary antibody was goat anti-rabbit Alexa-488 (1∶500; Invitrogen); this was applied for 2 hours and counterstained with propidium iodide.

### Analyses of placode size, apoptosis, proliferation, and total number of cells

For quantification of placode size, wild-type and *Spry2^−/−^* tongues between E13 and E14 were stained with anti-E-Cadherin antibody and the area measured using ImageJ software. Apoptosis in the CV placode at E12.5 was measured using the *In situ* Cell Death Detection kit (Roche) following manufacturer's protocol. Proliferating cells were identified by anti-PCNA immunofluorescence staining or injection of 1 mg BrdU for 2 hours followed by staining with anti-BrdU antibody (Invitrogen). Anti-PCNA stained and total (i.e. DAPI-stained) number of cells were counted using ImageJ software and presented as a percentage of proliferating cells. The total number of cells in the CV placode was quantified from confocal images of E-cadherin stained sections using Volocity5 software (Improvision).

### qPCR

qPCR reactions were performed using the GoTaq qPCR Master Mix (Promega) in a Mastercycler Realplex (Eppendorf). All primers were designed using PerlPrimer3 software [56]; sequences are available upon request. qPCR conditions were as follows: 95°C, 2 minutes; 40 cycles at 95°C,15 seconds; 58°C,15 seconds; 68°C, 20 seconds; followed by a melting curve gradient. Expression levels for the genes of interest were normalized to levels of *L19* and are presented as levels relative to wild-type.

### Organ culture

Developing tongues at E11.5 and E12.5 were isolated and cultured on 0.45 µm Millicell-HA membranes (Millipore) in F12/DMEM medium (GIBCO/Invitrogen) containing 1% FBS, 2% B27 culture supplement (GIBCO/Invitrogen), and antibiotics. FGF signaling was inhibited by addition of 25 µM SU5402 suspended in DMSO (Calbiochem), and an equivalent volume of DMSO was added to control wells. After 3 days in culture, the tongues were fixed in 4% paraformaldehyde for 2 h and analyzed.

### Microscopy

Fluorescent and bright field images were taken using a Leica DM5000B with a Leica DFC500 camera. For confocal images, a Leica SP5 Confocal was used.

### Sample size, penetrance, and statistical analysis

Unless otherwise noted, all experiments were performed independently in triplicate on at least three different specimens (N≥3), and when applicable, presented as an average ± standard deviation. Unpaired Student t-test was used to determine p-values and p<0.01 was deemed to be significant. The CVP phenotype observed in *Spry2* null mice was 100% penetrant (n>12); the loss of CVP in *Fgf10* null mice was observed in 100% of the mice (n = 6), however, there were indications of small trenches or invaginations (although absence of innervation) in 66% of the embryos; the rescue of the single CVP in the double *Fgf10*;*Spry2* null mice was 100% penetrant (n = 4); the presence of multiple (i.e. ≥3) CVPs in *Spry1*;*Spry2* null mice was 86% penetrant (n = 6). The Fisher exact probability test was used to determine the p-values of the tongue culture experiments.

## Supporting Information

Figure S1 CVP taste buds are innervated and are comprised of three types of taste receptor cells. Cartoons depict the sagittal views of figure. (A-F) Immunofluorescence staining (green) in wild-type and *Spry2* ^−/−^ mice show the presence of the three taste receptor cell types in the CVPs. Arrowheads point to the two CVPs in *Spry2* ^−/−^ mice. Scale bar, 100 µm. (G,H) β3-tubulin immunofluorescence staining shows the innervation of both CVPs in sagittal sections in tongues of *Spry2* ^−/−^ mice. Scale bar, 10 µm.
(PDF)

Figure S2 Innervation and placode size of the CVP. (A-I) H&E staining of coronal sections of the developing CVP between E12.5 and E13.5. Scale bar, 50 µm. (J) Quantification of the placode size between E13 to E14. **, p<0.001.
(PDF)

Figure S3 Expression levels of various genes and quantification of cell death in the CV placode at E12.5. (A) Total RNA was extracted from the presumptive CVPs in wild-type and *Spry2* ^−/−^ littermates. The expression levels of various genes of interest were assayed by qPCR. *, p<0.01; **, p<0.001. (B-D) TUNEL assay was performed to quantify apoptotic cells. DNase-treated sections from wild-type served as positive controls. Scale bar, 20 µm.
(PDF)

Figure S4 Inhibition of FGF signaling leads to the absence of CVP development *in vitro*. (A-E') Tongues were isolated from mice at E11.5 (A-B') and E12.5 (C-E'), grown in the absence and presence of an inhibitor of FGF signaling (SU5402) for 3 days *in vitro*, and immunostained using anti-E-cadherin. In tongues from E11.5, there was an absence of CVP with SU5402 treatment (A-B'). In E12.5 tongues, there was either an absence of CVP (D,D') or the presence of a malformed CVP (E,E') relative to controls (Co; C,C'). Scale bars, 50 µm. (F) A summary of observations is presented with p-values calculated using the Fisher exact probability test.
(PDF)

Figure S5 β3-tubulin immunofluorescence staining demonstrates the innervation of multiple CVPs in tongues of DKO mice. Scale bar, 20 µm.
(PDF)

Figure S6 Summary of the variation in CVP number in the mammalian taxa. (A) Perturbation of FGF signaling leads to effects on CVP number in mice ranging from zero to three CVPs. The speculative levels of FGF signaling are correlated with the number of CVPs in the anterior-posterior orientation observed in various mammalian species including the siamang, gorilla, chimpanzee, and lemur. (B) Variation in mammalian CVP number. Common species are listed for each clade. The red numbers represent the typical number of CVPs with bracketed numbers showing the range of numbers observed.
(PDF)

Figure S7 Analysis of the variation in mammalian CVP number. Numbers represent the number of CVPs. The clades are color-coded: Brown, Monotremata; Orange, Metatheria; Dark blue, Afrotheria; Yellow, Xenarthra; Purple, Glires; Red, Archonta; Green, Lipotyphla; Pink, Chiroptera; Black, Ferae; Light Blue, Euungulata. The number of CVPs is color-coded: Red, <3; Black,  = 3; Blue, 4–10; Green, >10.
(PDF)
