# Supplementary figures and images for: FGF Signaling Regulates the Number of Posterior Taste Papillae by Controlling Progenitor Field Size
Source: PLoS Genet. 2011 Jun 2;7(6):e1002098. doi: 10.1371/journal.pgen.1002098 (PMC3107195; doi:10.1371/journal.pgen.1002098)

Figure S1

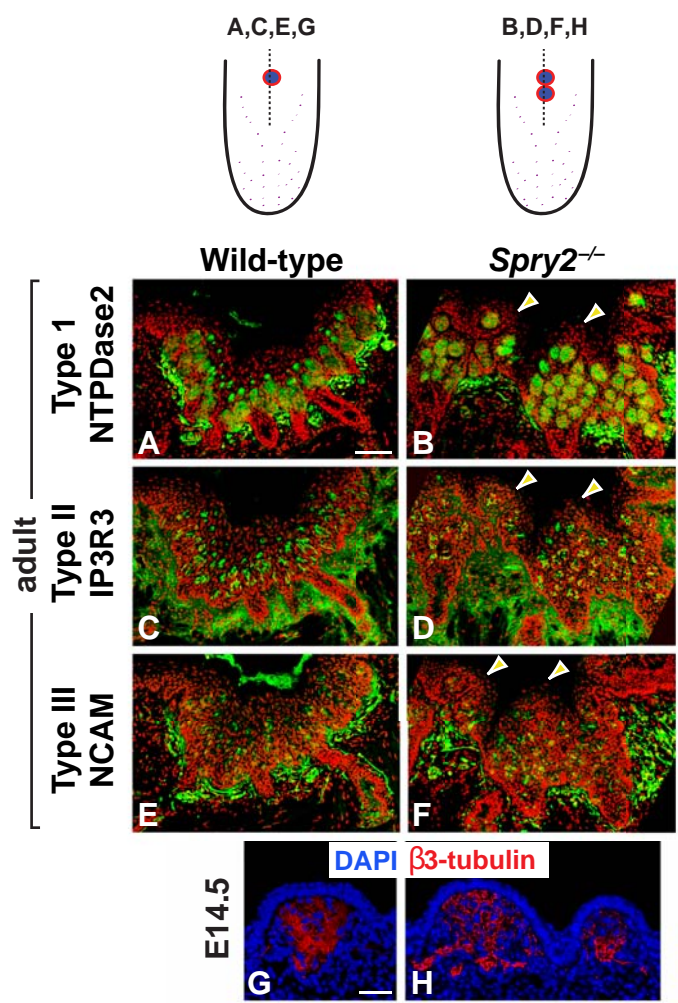

Supplement: Figure S1 — CVP taste buds are innervated and are comprised of three types of taste receptor cells. Cartoons depict the sagittal views of figure. (A-F) Immunofluorescence staining (green) in wild-type and Spry2 −/− mice show the presence of the three taste receptor cell types in the CVPs. Arrowheads point to the two CVPs in Spry2 −/− mice. Scale bar, 100 µm. (G,H) β3-tubulin immunofluorescence staining shows the innervation of both CVPs in sagittal sections in tongues of Spry2 −/− mice. Scale bar, 10 µm. (PDF) [file pgen.1002098.s001.pdf]

Figure S2

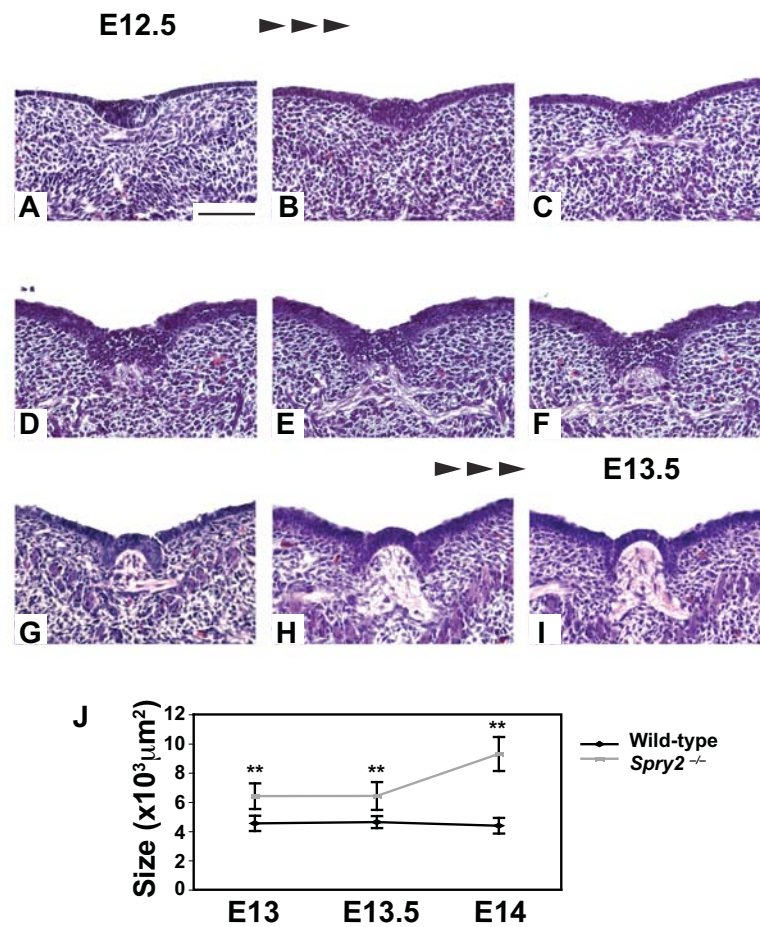

Supplement: Figure S2 — Innervation and placode size of the CVP. (A-I) H&E staining of coronal sections of the developing CVP between E12.5 and E13.5. Scale bar, 50 µm. (J) Quantification of the placode size between E13 to E14. **, p<0.001. (PDF) [file pgen.1002098.s002.pdf]

Figure S3

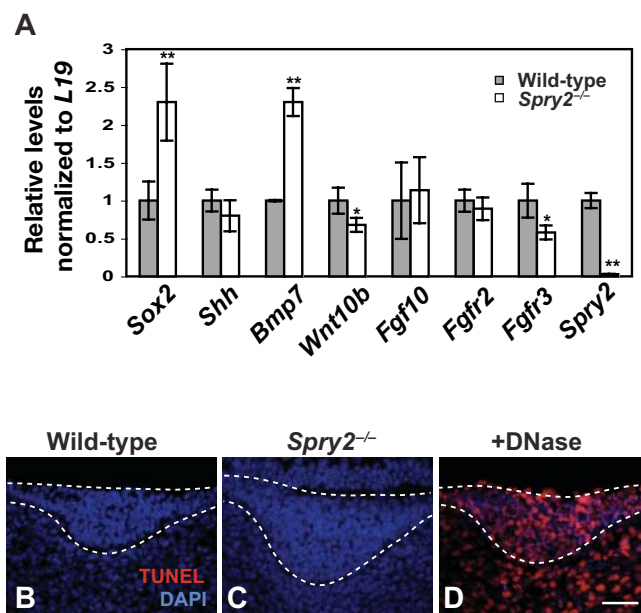

Supplement: Figure S3 — Expression levels of various genes and quantification of cell death in the CV placode at E12.5. (A) Total RNA was extracted from the presumptive CVPs in wild-type and Spry2 −/− littermates. The expression levels of various genes of interest were assayed by qPCR. *, p<0.01; **, p<0.001. (B-D) TUNEL assay was performed to quantify apoptotic cells. DNase-treated sections from wild-type served as positive controls. Scale bar, 20 µm. (PDF) [file pgen.1002098.s003.pdf]

Figure S4

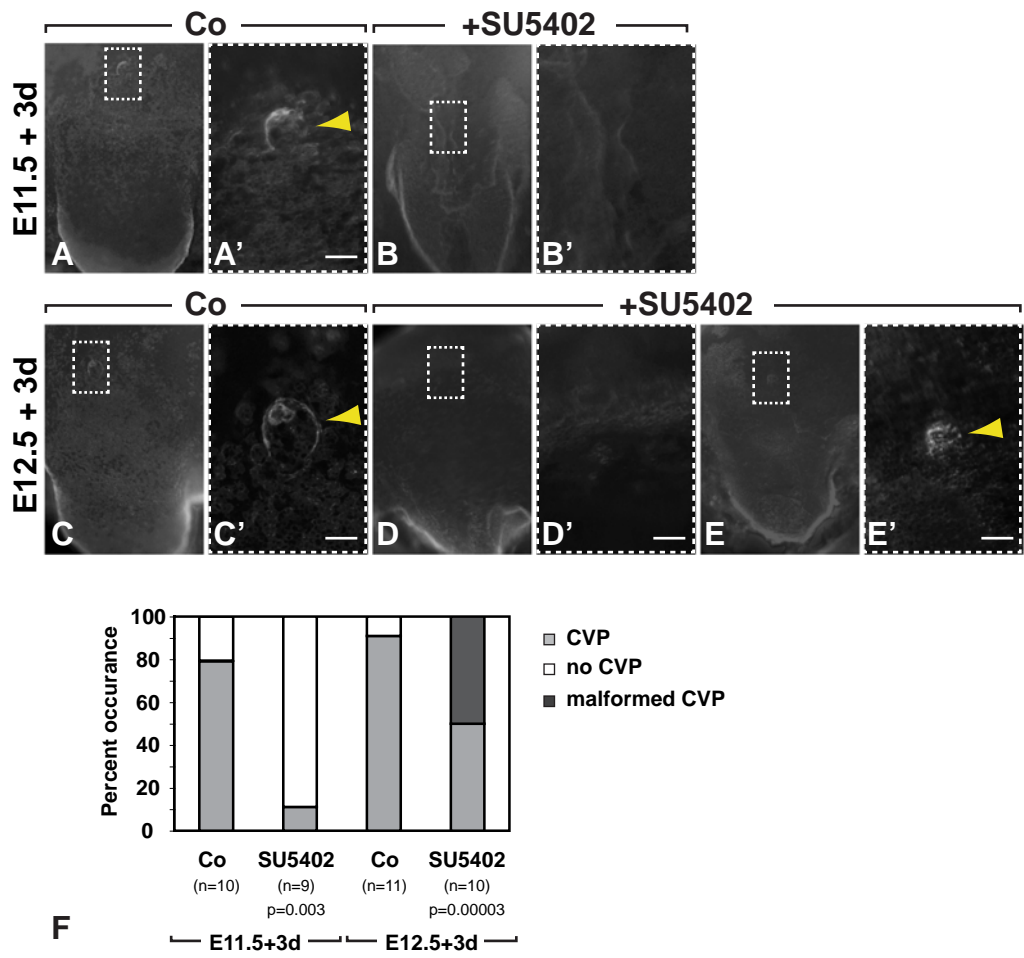

Supplement: Figure S4 — Inhibition of FGF signaling leads to the absence of CVP development in vitro. (A-E') Tongues were isolated from mice at E11.5 (A-B') and E12.5 (C-E'), grown in the absence and presence of an inhibitor of FGF signaling (SU5402) for 3 days in vitro, and immunostained using anti-E-cadherin. In tongues from E11.5, there was an absence of CVP with SU5402 treatment (A-B'). In E12.5 tongues, there was either an absence of CVP (D,D') or the presence of a malformed CVP (E,E') relative to controls (Co; C,C'). Scale bars, 50 µm. (F) A summary of observations is presented with p-values calculated using the Fisher exact probability test. (PDF) [file pgen.1002098.s004.pdf]

Figure S5

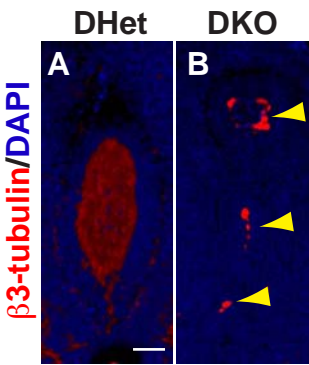

Supplement: Figure S5 — β3-tubulin immunofluorescence staining demonstrates the innervation of multiple CVPs in tongues of DKO mice. Scale bar, 20 µm. (PDF) [file pgen.1002098.s005.pdf]

Figure S6

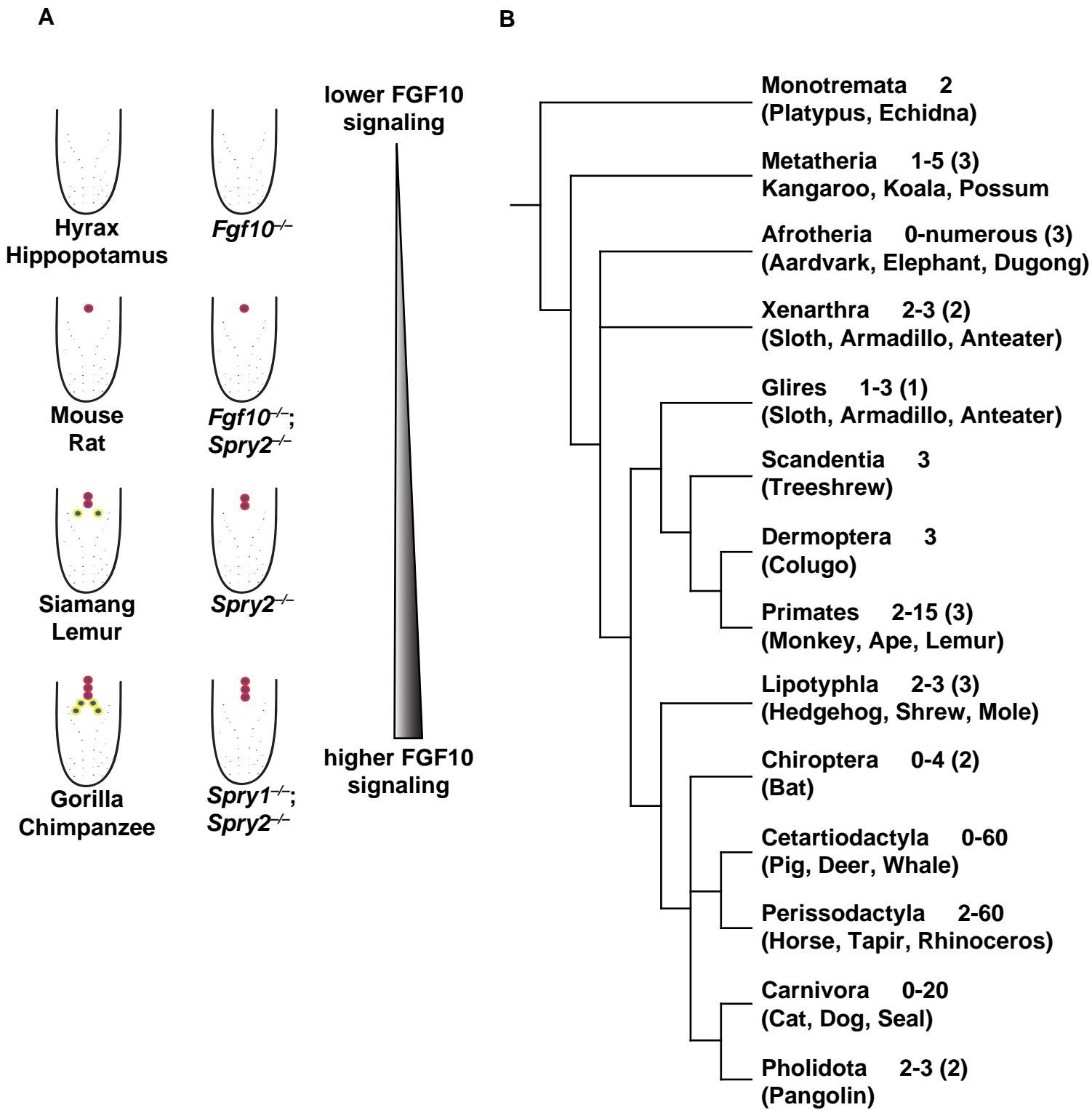

Supplement: Figure S6 — Summary of the variation in CVP number in the mammalian taxa. (A) Perturbation of FGF signaling leads to effects on CVP number in mice ranging from zero to three CVPs. The speculative levels of FGF signaling are correlated with the number of CVPs in the anterior-posterior orientation observed in various mammalian species including the siamang, gorilla, chimpanzee, and lemur. (B) Variation in mammalian CVP number. Common species are listed for each clade. The red numbers represent the typical number of CVPs with bracketed numbers showing the range of numbers observed. (PDF) [file pgen.1002098.s006.pdf]

**Figure S7**

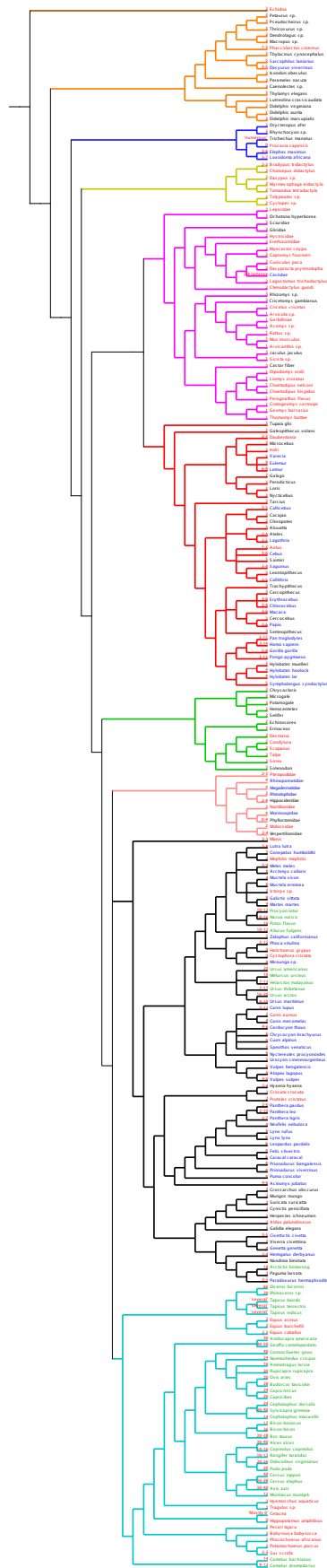

Supplement: Figure S7 — Analysis of the variation in mammalian CVP number. Numbers represent the number of CVPs. The clades are color-coded: Brown, Monotremata; Orange, Metatheria; Dark blue, Afrotheria; Yellow, Xenarthra; Purple, Glires; Red, Archonta; Green, Lipotyphla; Pink, Chiroptera; Black, Ferae; Light Blue, Euungulata. The number of CVPs is color-coded: Red, <3; Black, = 3; Blue, 4–10; Green, >10. (PDF) [file pgen.1002098.s007.pdf]
